# Fourier ptychographic microscopy for filtration-based circulating tumor cell enumeration and analysis

**DOI:** 10.1117/1.JBO.19.6.066007

**Published:** 2014-06-20

**Authors:** Anthony Williams, Jaebum Chung, Xiaoze Ou, Guoan Zheng, Siddarth Rawal, Zheng Ao, Ram Datar, Changhuei Yang, Richard Cote

**Affiliations:** a University of Miami, Miller School of Medicine, Department of Pathology, 1501 NW 10th Avenue BRB 742, Miami, Florida 33136; b University of Miami, Dr. John T. Macdonald Foundation Biomedical Nanotechnology Institute (BioNIUM), 1501 NW 10th Avenue BRB 714, Miami Florida 33136; c California Institute of Technology, Departments of Electrical Engineering, Bioengineering, and Medical Engineering, 1200 East California Boulevard MC 136-93, Pasadena, California 91125

**Keywords:** metastasis, circulating tumor cells, Fourier ptychographic microscopy, microfilter device

## Abstract

Circulating tumor cells (CTCs) are recognized as a candidate biomarker with strong prognostic and predictive potential in metastatic disease. Filtration-based enrichment technologies have been used for CTC characterization, and our group has previously developed a membrane microfilter device that demonstrates efficacy in model systems and clinical blood samples. However, uneven filtration surfaces make the use of standard microscopic techniques a difficult task, limiting the performance of automated imaging using commercially available technologies. Here, we report the use of Fourier ptychographic microscopy (FPM) to tackle this challenge. Employing this method, we were able to obtain high-resolution color images, including amplitude and phase, of the microfilter samples over large areas. FPM’s ability to perform digital refocusing on complex images is particularly useful in this setting as, in contrast to other imaging platforms, we can focus samples on multiple focal planes within the same frame despite surface unevenness. In model systems, FPM demonstrates high image quality, efficiency, and consistency in detection of tumor cells when comparing corresponding microfilter samples to standard microscopy with high correlation ($R^{2}=0.99932$). Based on these results, we believe that FPM will have important implications for improved, high throughput, filtration-based CTC analysis, and, more generally, image analysis of uneven surfaces.

## 1 Introduction

Metastatic disease accounts for 90% of cancer-related mortality, and is the most important determinant in clinical management of patients with cancer. As a result, circulating tumor cells (CTCs) in peripheral blood have emerged in recent years as a valuable biomarker with strong potential to improve prognosis and diagnosis of recurrence. Assaying for CTCs requires only a simple, minimally invasive blood draw, providing a unique opportunity for repeated sampling in patients to monitor both metastatic disease as well as therapeutic response in real time. Thus, the enumeration of CTCs with respect to progression-free survival, overall survival, and therapeutic response has been widely reported on in a number of solid tumor malignancies.^1^^–^^6^

Currently, isolation of CTCs by density gradient centrifugation,^7^^,^^8^ indirect detection of CTCs by RT-PCR,^9^^–^^11^ and affinity-based capture of CTCs using cell surface markers specifically expressed by malignant cells,^1^^–^^6^^,^^12^ are the strategies most commonly used to identify and isolate CTCs. However, current strategies for CTC analysis each have limitations.^13^^,^^14^ In addition, size-based isolation of CTCs from whole blood has been attempted since 1960s,^15^ and has been revisited more recently. Utilizing the well-known characteristic that the malignant cells are larger than surrounding normal blood cells, CTCs are isolated by using microfilters fabricated with a defined pore size, which allow for the passage of smaller blood cells to pass while capturing larger CTCs.^15^^–^^22^ Where the sensitivity and efficiency of affinity-based CTC enrichment strategies rely primarily on tissue- and/or tumor-specific cell surface biomarkers with the potential for highly variable inter tumor expression, size-based enrichment technologies are “antigen expression-agnostic,” allowing analysis of CTCs in tumor types with low or no target antigen expression.

With the potential to overcome the limitations accompanying other platforms, size-based CTC enrichment strategies possess technical limitations of their own. The most significant of these limitations is that following sample processing, the surface of the microfilters becomes uneven with short, microscale modulations. Because captured cells are randomly dispersed throughout the microfilter, often times multiple CTCs are present on different focal planes. This technical limitation requires that the user must constantly change focus while viewing and imaging cells of interest on the microfilter, making sample analysis labor intensive, time consuming, and inefficient. Automated imaging systems have been developed and are widely available on a number of microscopy platforms, and could potentially alleviate these complications. However, such systems cannot readily be employed for CTC analysis using filtration-based technologies due to their inability to focus multiple areas with different focal planes within the same frame.

Further complicating this issue, when viewing microfilters under a microscope to identify CTCs, the entire filtration area must be viewed by systematically moving up and down the microfilter in columns (i.e., in y-axis plane), or side to side across the microfilter in rows (i.e., x-axis plane) manually using the stage manipulator knobs. In instances where the observer does not appropriately align adjoining columns and or rows on the microfilter, small areas where tumor cells may reside can escape the field of view (FOV) and fail to be counted, or single events can be counted more than once if adjoining columns or rows are aligned slightly overlapping each other. Without the ability to produce images for CTC analysis in an automated fashion, the potential for inter-operator variability and inconsistent analyses between users and collaborating institutions reviewing the same samples is dramatically increased. These technical limitations, taken together, prevent widespread analysis of CTCs using filtration-based CTC enrichment technologies.

To address these challenges, we present the adaptation of Fourier ptychographic microscopy (FPM) for the identification and enumeration of CTCs captured by size-based enrichment. The use of FPM allows for the rapid generation of continuous, high-resolution images over large areas of interest. Importantly, we also describe the ability to perform digital refocusing of images generated by FPM on a frame-by-frame basis, allowing us to create focused images of frames containing cells of interest in multiple focal planes, thus traversing the limitations in automated imaging of uneven surfaces produced by other commercially available technologies. Here, we present an assessment of our ability to analyze CTCs captured on a previously described membrane microfilter device developed by our group using FPM, evaluating the efficiency of CTC detection as well as the image quality of CTCs generated by FPM relative to standard microscopic analysis.

## 2 Membrane Microfilter Device for Circulating Tumor Cell Capture and Characterization

Among others,^16^^–^^20^ our group has developed a membrane microfilter device for the size-based isolation of CTCs in blood.^21^^,^^22^ Microfilters for CTC capture and analysis are fabricated using a precisely defined, stepwise photolithography process as previously described.^21^ Our technology provides the opportunity to perform molecular characterization of CTCs beyond their enumeration, a critical step toward a better understanding of the mechanisms involved in their release, hematogenous spread, and colonization of tissues at distant sites from the tumor origin. Although based on similar principles for CTC enrichment, the fundamental differences between our technology and other size-based technologies are (1) the material from which the filters are manufactured (i.e., parylene-C versus polycarbonate, respectively) and (2) the manner in which the pores are deposited onto the membrane, where pores are evenly and specifically dispersed on our device rather than randomly dispersed.^21^ We have evaluated the performance of the microfilter device in model systems and clinical samples, where in side-by-side comparison with the CellSearch platform, the microfilter device demonstrated superior sensitivity in both model systems and clinical blood samples.^22^

## 3 Principles of Fourier Ptychographic Microscopy

**Fig. 1:**
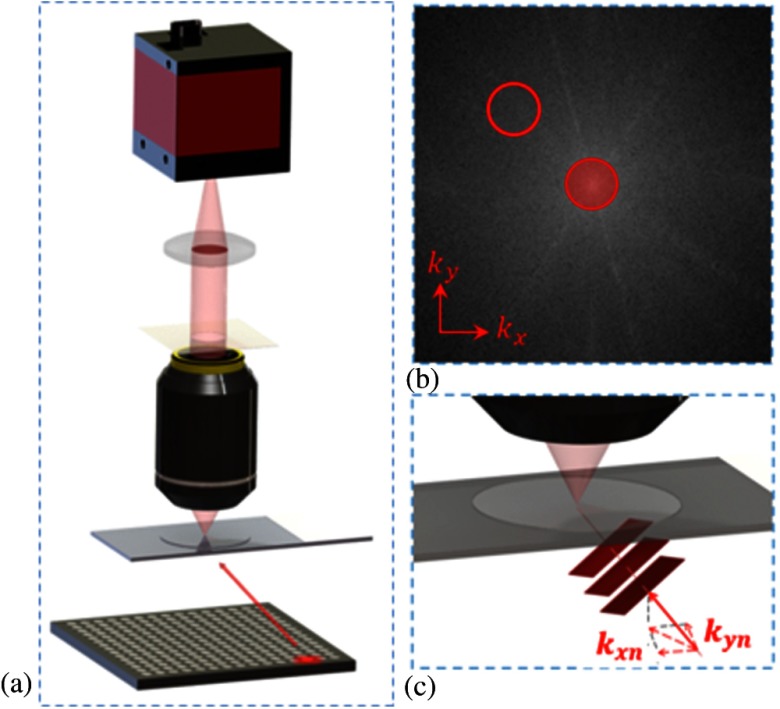
(a) The FPM setup consists of (from the bottom) an LED matrix for sample illumination, a microscope system with a 2× objective, and a camera connected to a computer. (b) The Fourier spectrum point of view. The center red subregion corresponds to the spatial frequency of the low-resolution image captured with plane waves with $k_{x}=k_{y}=0$. The off-center red subregion correlates to an oblique angle illumination with wavevector $\left(k_{x},k_{y}\right)$. (c) Light from an LED at an oblique angle corresponds to a plane wave with a k vector $\left(k_{x},k_{y}\right)$.

**Fig. 2:**
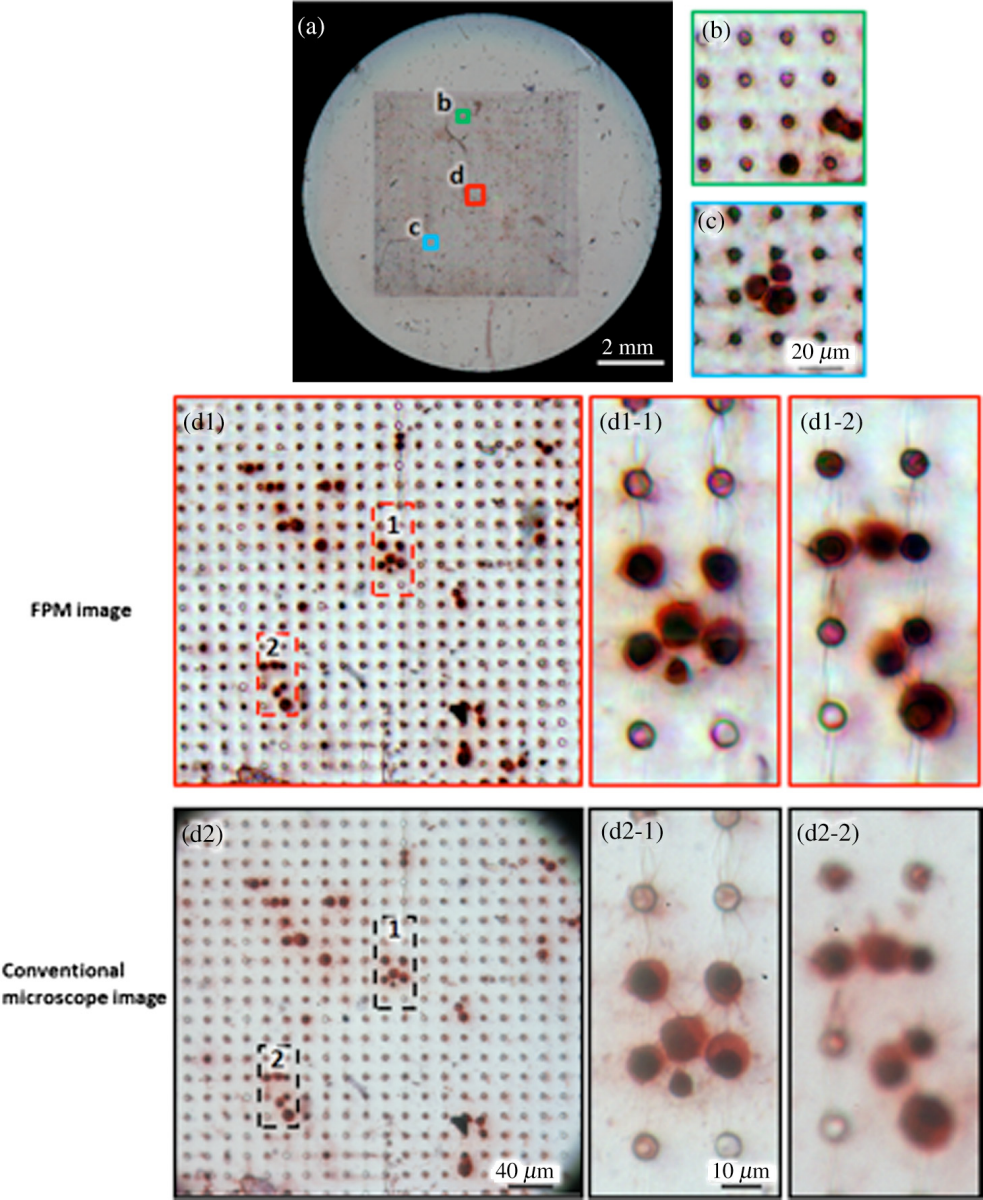
(a) Full field-of-view color image of the entire microfilter containing captured tumor cells by FPM. Magnified FPM images (b-d1) selected from different areas of the microfilter show detailed morphology of tumor cells, where all sections are well in focus because of the automatic EPRY-FPM program. (d2) A standard microscope (with 40× objective) image shows the corresponding region to (d1), but because of the uneven surface of the microfilter, subregions (d2-1) and (d2-2) cannot be focused simultaneously. Also, its field of view is limited when compared to (d2), as seen by the aperture’s outline at the edge of the image, in contrast to FPM’s wide field-of-view that can provide high-resolution images of the entire microfiltration area.

FPM is a method that can capture a wide field-of-view image with high resolution.^23^^,^^24^ The core principle of FPM is its ability to acquire high spatial frequency components of the sample by illuminating it with a plane wave at oblique angles. The FPM setup consists of the following components: a Olympus BX 41 microscope with 0.08 NA using a Plan APO 2× objective lens (Olympus, Center Valley, Pennsylvania), a KAI-29050 interline CCD camera with 5.5 *μ*m pixel size (Eastman Kodak, Rochester, New York) attached to a computer for image capturing and processing, and a square light-emitting diode (LED) array for illumination. The LED matrix contains 32×32 surface-mounted, full-color LEDs and adjacent LEDs are laterally separated by 4 mm. The full-color LED has central wavelengths of 632 nm (red), 532 nm (green), and 472 nm (blue), each offering a spatially coherent quasimonochromatic source with ∼20  nm bandwidth. When an LED on the matrix is activated, its light field incident on the sample can be approximated as a plane wave due to the large distance (∼8  cm) between the LED and the sample plane. The angular illumination can be characterized by its in-plane wavevector $\left(k_{x},k_{y}\right)$ within the coordinate system, as depicted in Fig. 1. Illuminating a sample with a plane wave of a wavevector in the space domain is equivalent to shifting the center of the sample’s frequency spectrum in the Fourier domain. Because the objective acts as a circular, low-pass filter in Fourier domain, each captured image carries information describing a shifted small subregion, which is geometrically defined by the pupil function of the microscope, of the sample’s frequency spectrum. Images are captured by the camera with single LEDs of one color activated in sequence acting as the light source, and a phase retrieval algorithm is used to stitch the subregions together in the Fourier domain to form a high-resolution complex image, which contains both amplitude and phase information. Red, green, and blue images are acquired separately by altering the color of the LED for each set of acquisitions. Single color channels are used individually for the entire image capturing and reconstruction process. Thus, the procedure is conducted a total of three times for red, green, and blue channels. These images are later combined to create a full-color image, such as the ones shown in Fig. 2. In our hands, each sample takes ∼3  min to capture and 10 min to reconstruct each of the three color channels, for a total of 39 min to produce a color image of the entire field. For sample analysis by the observer, the complex whole FOV image created by FPM is refocused and partitioned into a total of ∼300 tiles to be used for CTC identification on the entire field.

**Fig. 3:**
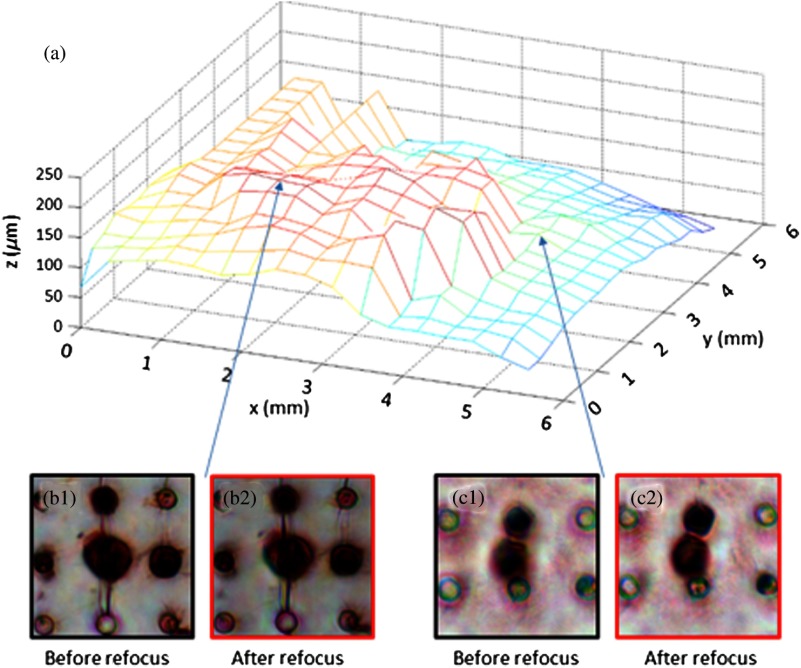
(a) A microfilter’s surface profile characterized by the pupil function recovered from EPRY-FPM algorithm. The focal plane of the objective is at 150 *μ*m. The maximum difference in-depth across the filter is about 250 *μ*m, which is within FPM’s refocusing capacity of 300 *μ*m. In this case, the captured microfilter image is sectioned into 17×17 tiles, and EPRY-FPM iteratively characterizes each tile’s defocus level in its high-resolution image reconstruction process. (b1)-(c2) Small subregions are extracted from two different surface levels, showing before and after refocusing by EPRY-FPM. (b1) is already very close to the focal plane, so there is only a minor improvement after refocusing, as in (b2). (c1) is not in the focal plane and is blurry. (c2) shows the refocused result.

Because of the uneven surface of the microfilter containing cells of interest, an image taken by a standard microscope suffers from defocus. As shown in Fig. 2, when some parts in the FOV are in focus [Figs. 2-(d1-1)], other parts can be blurry [Fig 2-(d1-2)]. Embedded pupil function recovery FPM (EPRY-FPM), a new phase retrieval algorithm developed by Ou et al., can automatically correct for this aberration.^25^ When EPRY-FPM stitches the subregions in the Fourier domain, it does so by iteratively recovering both the Fourier spectrum of the sample and the pupil function. Because of the pupil function, which contains the aberration of the lens system and the defocus caused by surface unevenness, the Fourier spectrum of the sample is separated during the EPRY-FPM process. Performing an inverse Fourier transformation on the sample’s Fourier spectrum results in an aberration-free, flattened image of the microfilter. As shown in Fig. 2-(d1), the defocus is corrected automatically and the components of the image shown in Figs. 2-(d1-1) and 2-(d1-2) are well focused. Because the algorithm also recovers the pupil function, the sample’s depth information can be obtained and graphed, as shown in Fig. 3(a). The graph shows the microfilter’s severe surface unevenness, which requires an imaging device with different focusing levels across the sample. FPM, with its refocusing capacity of up to 300 *μ*m, is able to image the entire microfilter in focus. Figures 3(b1) and 3(c1) show two different areas at different depth levels on the microfilter, with 3(b1) being in focus and 3(c1) being out of focus. After applying FPMs refocusing algorithm, both areas are brought sharply in focus, as shown in Figs. 3(b2) and 3(c2).

## 4 Methods

### 4.1 Cell Culture

The SKBR-3 breast cancer cell line was obtained commercially from the American Type Tissue Collection (Manassas, Virginia), thawed to room temperature, and placed into a T25 culture flask containing McCoy’s 5a culture media (Gibco, Carlsbad, California) supplemented with 10% fetal bovine serum (Mediatech, Manassas, Virginia) and 100 units per ml penicillin-streptomycin (Gibco, Carlsbad, California). At 80% confluency, the cells were harvested from the culture flask using 0.25% trypsin-EDTA (Gibco, Carlsbad, California), washed twice in 1x Hank’s Buffered Salt Solution (HBSS; Invitrogen, Carlsbad, California) and pelleted by centrifugation at 500×g.

### 4.2 Tumor Cell Counting and Seeding into Normal Donor Blood

SKBR-3 cell concentration per ml solution was obtained using the Sceptor 2.0 automated handheld cell counter (EMD Millipore, Billerica, Massachusetts), and the solution was then diluted to a final concentration of $1\times 10^{4}\text{ tumor cells}/\mathrm{ml}$. For experiments testing a higher number of tumor cells (i.e., >15 cells seeded) captured on the microfilter, the actual number of tumor cells seeded into blood must be carefully determined, as (a) there is the potential for high variability introduced into aliquots prepared by serial dilutions and (b) the process of pipetting tumor cells into the blood itself can be inconsistent from sample to sample. To account for potential variability, for each experimental replicate, the aliquot volume (i.e., 50 *μ*l) to be delivered from the diluted cell solution into the donor blood was placed onto a hemocytometer and manually counted 10 different times. From these manual counts, the average number of cells with standard deviation was calculated to more precisely determine the “actual” target number of cells to be recovered from the blood and identified by each respective microscopy technology. For two experiments testing the lower limit of tumor cell capture, to remove the Poisson sampling variability that is introduced by serial dilution preparation, tumor cells were manually micropipetted while being viewed under a dissecting microscope and introduced directly into the donor blood samples.

### 4.3 Tumor Cell Capture by the Microfilter Device

In preparation for filtration, individual microfilters are cut away from a wafer containing multiple microfilters and placed into an acrylic housing cassette where each is sandwiched between two thin slabs of polydimethylsiloxane, and clamped at each end to form an airtight seal.^26^ 10 ml whole blood samples, collected into anticoagulant vaccutainer tubes, are diluted 1∶1 in 1× phosphate buffered saline (PBS; Invitrogen, Carlsbad, California) and 1/10 volume of 10% neutral buffered formalin is added (NBF; VWR International, Radnor, Pennsylvania) to the diluted blood for a final volume of 20 ml, at a final concentration of 1% NBF. Diluted, formalin-fixed blood samples are placed on a rocker for 10 min at room temperature, and are passed through the microfilter by a syringe fixed onto a luerlock on top of the acrylic housing cassette at a constant flow rate of 200  ml/hr using a motorized syringe pump. Following filtration, microfilters containing CTCs are disengaged from the filtration cassette and placed onto a glass microscope slide for downstream molecular analysis. Our method for CTC identification is a double marker immunohistochemistry (IHC) protocol (described later in Sec. 4.4) that includes a pan-cytokeratin (CK) antibody for identification of epithelial tumor cells and a CD45 antibody for simultaneous positive selection of tumor cells and negative selection of large, and tumor blood cells, respectively. The entire 6×6  mm area of the microfilter is viewed under a microscope, and CTCs are identified as large (typically 15–40 *μ*m diameter), nucleated, CK+/CD45- events with morphological criteria consistent with malignant cells.

### 4.4 Immunohistochemistry on Tumor Cells Captured by the Microfilter Device

Following cell seeding and filtration, the microfilters were placed into 24-well culture dishes to facilitate simultaneous immunolabeling of multiple microfilters (instead of glass microscope slides) and washed for 10 min in 1x tris buffered saline (TBS; Biocare Medical, Concord, California). The filters were rehydrated with washes in decreasing concentrations of alcohol (100%, 90%, and 70%) for 6 min each wash. To quench any endogenous perioxidase activity, the microfilters were washed in 0.03% $H_{2}O_{2}$ + methanol for 20 min. Following the perioxidase quench, the microfilters were washed with deionized $H_{2}O$, and nonspecific reactivity with primary antibodies was blocked with buffer containing 5% normal goat serum, 1% BSA, and 0.3% Triton X-100 in 1x TBS for 30 min. Rabbit antihuman CK (1∶300; DAKO, Carpinteria, California) and mouse antihuman CD45 (Ready-to-use; DAKO, Carpinteria, California) were cocktailed together and incubated with the microfilters overnight at room temperature. The microfilters were washed in 1x TBS for 10 min, and then incubated with MACH 2 secondary antibody buffer (Biocare Medical, Concord, California), containing both conjugated goat antirabbit alkaline phosphatase (AP) and goat antimouse horseradish perioxidase (HRP) activity, for 30 min. Microfilters were washed in 1x TBS for 10 min, then incubated with 3’, 3’-diaminobenzidine (DAB; Biocare Medical, Concord, California), reactive with HRP, for 5 min to form a brown precipitate reporting antiCD45 reactivity. Microfilters were washed in deionized $H_{2}O$, and then incubated with Warp Red (Biocare Medical, Concord, California), reactive with AP, for 7 min to form a red precipitate reporting antiPan CK reactivity. Microfilters were washed with deionized $H_{2}O$, incubated with CAT hematoxylin (Biocare Medical, Concord, California) 3 min for nuclear visualization, Tacha’s Bluing Reagent (Biocare Medical, Concord, California) for 3 min, dehydrated with increasing concentrations of alcohol (70%, 90%, and 100%), washed with xylene, and coverslipped using mounting medium (Richard Allan Scientific, Waltham, Massachusetts). To verify antibody specificity, cytospin slides were prepared using a mixture of SKBR-3 and peripheral blood mononuclear cells (1∶4), enriched by gradient-based centrifugation from a normal donor, and used as positive controls.

## 5 Circulating Tumor Cell Analysis Using Fourier Ptychographic Microscopy Versus Standard Microscopy in Model Systems

SKBR-3 breast cancer cells were seeded into 5 ml of whole blood from a normal, healthy donor at various concentrations, processed by the microfilter device, and labeled with CK and CK45 by double marker IHC on glass microscope slides. A total of 11 replicates of this experiment were performed. Nine normal donor blood samples were seeded with a range of tumor cells (15 to ∼800), representing the lower and upper limits of CTCs typically identified from clinical blood samples using the microfilter device. As a negative control, two normal donor blood samples containing no tumor cells were processed by the microfilter device and analyzed microscopically using both technologies. In all samples tested, captured tumor cells were enumerated first by “standard microscopy”—Axio Imager M1 with an Apochromat 20×/0.8 NA objective lens (Carl Zeiss Microscopy LLC, Thornwood, New York) and the tumor cell counts were compared to enumeration done by FPM in corresponding samples.

**Fig. 4:**
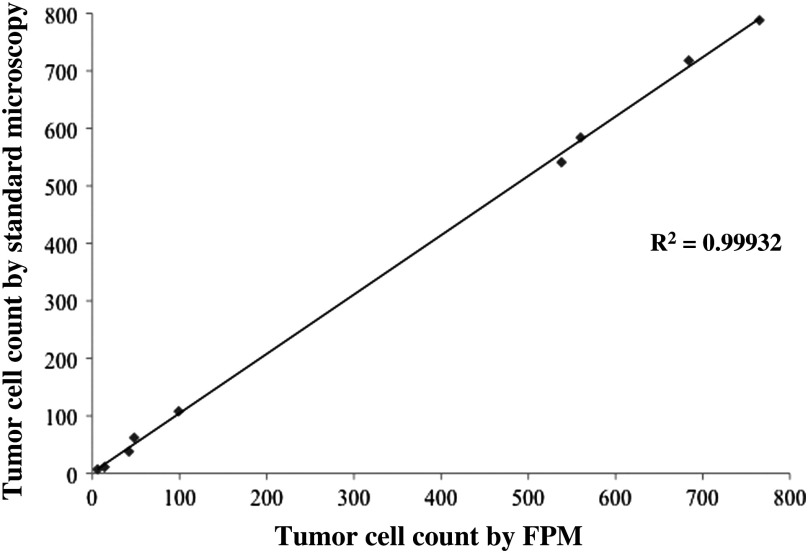
Graph demonstrating the correlation between tumor cell count by standard microscopy (y-axis) and tumor cell count by FPM (x-axis) in corresponding microfilter samples, where each data point represents a single trial with tumor cells enumerated by both methods.

Table 1 demonstrates the comparison of tumor cells identified by both microscopy technologies among corresponding samples captured by the microfilter device. The primary objective of this comparison was to evaluate the ability of FPM versus our standard method of microscopy to produce high-resolution images that can be used to accurately and efficiently detect tumor cells captured by the microfilter device. No tumor cells were detected in the negative control samples by either technology (Table 1), indicating that the images produced by FPM were suitable for the identification of specific biomarker reactivity and evaluation of morphologic criteria, both important parameters used to differentiate tumor cells from nucleated, tumor blood cells. Tumor cell identification rates on microfilters by standard microscopy (85.7%±6.68%; Table 1) are consistent with recovery rates previously reported using our CTC enrichment system in model system experiments,^21^^,^^22^ and are also consistent with tumor cell identification rates in corresponding microfilter samples evaluated by FPM (88.8%±8.3%; Table 1). Further, a Pearson’s correlation between cells identified by both technologies on corresponding microfilter samples was conducted to evaluate the consistency of tumor cell identification by FPM relative to standard microscopy. As demonstrated in Fig. 4, the $R^{2}$ for tumor cells identified in corresponding microfilter samples was 0.99932, indicating a strong correlation between tumor cells counted by both technologies. This further demonstrates FPM as a suitable imaging method that provides comparable accuracy in tumor cell detection and enumeration to standard microscopy.

**Table 1:** Table comparing detection of tumor cells seeded into normal donor blood by FPM and standard microscopy.

Tumor cells seeded	Tumor cell count by standard microscopy	Tumor cell count by FPM	% Recovery by standard microscopy	% Recovery by FPM
804 ±64	684	718	85.1% (78.8%–92.4%)	89.3% (82.0%–97.0%)^++^
582± 45	538	541	92.5% (85.8%–100%)	92.9% (86.2%–100%)^++^
812±72	765	788	94.2% (86.5%–100%)	97.0% (89.1%–100%)^++^
128 ± 14	99	108	77.3% (69.7%–86.8%)	84.3% (76.1%-94.7%)^++^
617±68	560	584	90.7% (81.8%–100%)	94.7% (85.3%–100%)^++^
*10	6	7	60%	70%^++^
61 ± 8	48	62	78.7% (69.6%–90.6%)	100% (89.9%–100%)^++^
55 ± 10	42	38	76.4% (64.6%–93.3%)^+^	69.1% (58.4%–84.6%)
*15	14	11	93.30%^+^	73.30%
0	0	0	0%	0%
0	0	0	0%	0%

Note: % recovered = (tumor cell count by FPM or standard microscopy/tumor cells seeded) × 100%. For each count observed, the upper and lower limits, as defined by the standard deviation in the number of cells seeded, are included in parentheses. % Recovery values labeled with “+” indicate trials where % recovery by standard microscopy > % recovery by FPM. % Recovery values labeled with “++” indicate trials where % recovery by standard microscopy < % recovery by FPM. Rows labeled with an asterisk indicate trials where tumor cells were micropipetted under observation with a dissecting microscope and directly introduced into normal donor blood, and thus have no standard error caused by serial dilution preparation.

## 6 Discussion

The primary aim of this paper is to evaluate whether the FPM can provide images that are suitable for CTC counting with comparable accuracy to a skilled microscopist operating a standard microscope. The near equivalence in accuracy that we found here is assuring and positive. As described earlier, we have highlighted the potential for errors in under or over counting CTCs on microfilters using standard microscopy. In contrast, FPM produces continuously tiled images composed of the entire filtration area with no significant error. Thus, the risk for missing or over counting small regions of view between rows or columns of the filtration area is removed by FPM. In the hands of a less skilled user, we can expect better counting accuracy for the FPM versus the standard microscope. Finally, it is worth noting that the examination of the slide under the standard microscope is laborious and highly manual. Digitization of the slide by FPM can allow for CTC counting in a more comfortable setting for the user in the near term, and allow for automatic counting in the future when appropriate algorithms have been developed.

As conventional whole slide imaging (WSI) systems allow for slide digitization as well, it is definitely worth discussing the relative merits of the FPM versus the conventional WSI scanning methods. Some of the advantages held by the FPM include simplified and robust system design due to the nonmechanical scanning approach, and the potential advantage in scan speed as the scan method is entirely optical. However, the strongest advantage that FPM has over conventional WSI systems for imaging the microfilters lies in the fact that FPM’s refocusing ability is very well suited to tackle the uneven nature of the microfilters. While certain conventional WSI systems are able to dynamically adjust their focal plane as they scan microscope slides, the focal plane at any given FOV through their objective is still fixed. As we can see from the standard microscopy images demonstrated in Figs. 2-(d2-1) and 2-(d2-2), only portions of the microfilter can be focused at any one time, despite the use of a 40X objective lens with a restrictive FOV. As shown, the FPM can nimbly adjust its focal plane, so that all parts of the sample are in focus.

Following this line of thought, we can also imagine that one of the ways conventional WSI systems can match this FPM advantage would be to exhaustively collect a z-stack of images at z-increments equal to the depth of field of the native objective. The data can be provided to the user to pick the right focal plane to examine each part of microfilter as desired. However, the data size associated with such an imaging method would be very significant. Using our current experiment microfilter as an example (desired FOV of 6-mm diameter, resolution of 0.78 *μ*m, objective’s depth of field of ∼5  μm, and a total sample height variation of 250 *μ*m), the total amount of data that a conventional WSI system would have to collect for a color image would be equal to ∼8.9  gigapixels, one-third of which is assigned to each of the RGB channels. In comparison, the FPM would have to collect ∼1.4  gigapixels worth of data for a color image and titrate that to a final image that contains ∼360  megapixels that can be refocused within a depth of field of 300 *μ*m. Additionally, each FPM image pixel contains both amplitude and phase information, while a standard WSI file is strictly an intensity (amplitude) map.

A potential advantage for the continued use of standard microscopy for the identification and molecular characterization of CTCs is the ability to perform immunofluorescent (IF) analyses using the microfilter. The use of secondary antibodies conjugated to specific fluorophores with narrow and intense spectra profiles enables the performance of downstream techniques, such as fluorescent *in situ* hybridization (FISH) and multimarker characterization of CTCs by IF. The FPM system used in this study is only functional for the conjugation of antibodies to secondary chromagens in the visible spectrum by brightfield microscopy. However, the FPM system can be readily modified to provide low-resolution fluorescence imaging capability. We simply have to insert an appropriate filter into the system and incorporate a sufficiently bright excitation light source. The resolution of such fluorescence images would be determined by the objective employed by the FPM. If the current FPM system was modified for fluorescence imaging, the fluorescence image resolution will be equal to ∼4  μm. At this resolution, the modified FPM would be able to identify which cells are fluorescing. Future studies using the FPM for CTC analysis will make attempts to provide low-resolution fluorescence imaging capability, which could potentiate the ability to view multiple fluorescently labeled biomarkers on CTCs in a multiplexed fashion.

## 7 Conclusion

Ours and several other groups have demonstrated size-based isolation by microfiltration to be a promising and efficient method for CTC enrichment. However, technical issues associated with postenrichment analysis using these technologies present limitations that could hinder advances in molecular characterization of CTCs and widespread use. Herein, we present FPM as a means to overcome these technical barriers, producing continuous, high resolution, and automated images of the entire filtration area. Where other automated imaging systems are limited, the ability of FPM to perform digital refocusing of each image by a phase-retrieval algorithm is a critical innovation of our system. Beyond the identification and characterization of CTCs using filtration-based technologies, FPM holds the potential for use in many other biomedical applications, such as immunohistochemistry in histology and cytology, as well as others that can benefit from a high resolution, wide FOV digital imaging technique with an automatic aberration correction.
